# Epidemiology of malaria among pregnant women during their first antenatal clinic visit in the middle belt of Ghana: a cross sectional study

**DOI:** 10.1186/s12936-020-03457-5

**Published:** 2020-10-23

**Authors:** David Kwame Dosoo, Daniel Chandramohan, Dorcas Atibilla, Felix Boakye Oppong, Love Ankrah, Kingsley Kayan, Veronica Agyemang, Dennis Adu-Gyasi, Mieks Twumasi, Seeba Amenga-Etego, Jane Bruce, Kwaku Poku Asante, Brian Greenwood, Seth Owusu-Agyei

**Affiliations:** 1 Kintampo Health Research Centre, Ghana Health Service, PO Box 200, Kintampo, Ghana; 2 Department of Disease Control, Faculty of Infectious and Tropical Diseases, London School of Hygiene and Tropical Medicine, London, UK; 3 Institute of Health Research, University of Health and Allied Sciences, Ho, Ghana

**Keywords:** Malaria parasitaemia, Anaemia, Prevalence, Risk factors, Antenatal clinic

## Abstract

**Background:**

Malaria during pregnancy may result in unfavourable outcomes in both mothers and their foetuses. This study sought to document the current burden and factors associated with malaria and anaemia among pregnant women attending their first antenatal clinic visit in an area of Ghana with perennial malaria transmission.

**Methods:**

A total of 1655 pregnant women aged 18 years and above with a gestational age of 13–22 weeks, who attended an antenatal care (ANC) clinic for the first time, were consented and enrolled into the study. A structured questionnaire was used to collect socio-demographic and obstetric data and information on use of malaria preventive measures. Venous blood (2 mL) was collected before sulfadoxine-pyrimethamine administration. Malaria parasitaemia and haemoglobin concentration were determined using microscopy and an automated haematology analyser, respectively. Data analysis was carried out using Stata 14.

**Results:**

Mean age (SD) and gestational age (SD) of women at enrolment were 27.4 (6.2) years and 16.7 (4.3) weeks, respectively. Overall malaria parasite prevalence was 20.4% (95% CI 18.5–22.4%). Geometric mean parasite density was 442 parasites/µL (95% CI 380–515). Among women with parasitaemia, the proportion of very low (1–199 parasites/µL), low (200–999 parasites/µL), medium (1000–9999 parasites/µL) and high (≥ 10,000 parasites/µL) parasite density were 31.1, 47.0, 18.9, and 3.0%, respectively. Age ≥ 25 years (OR 0.57, 95% CI 0.41–0.79), multigravid (OR 0.50, 95% CI 0.33–0.74), educated to high school level or above (OR 0.53, 95% CI 0.33–0.83) and in household with higher socio-economic status (OR 0.34, 95% CI 0.21–0.54) were associated with a lower risk of malaria parasitaemia. The prevalence of anaemia (< 11.0 g/dL) was 56.0%, and the mean haemoglobin concentration in women with or without parasitaemia was 9.9 g/dL or 10.9 g/dL, respectively.

**Conclusion:**

One out of five pregnant women attending their first ANC clinic visit in an area of perennial malaria transmission in the middle belt of Ghana had *Plasmodium falciparum* infection. Majority of the infections were below 1000 parasites/µL and with associated anaemia. There is a need to strengthen existing malaria prevention strategies to prevent unfavourable maternal and fetal birth outcomes in this population.

## Background

Despite efforts aimed at controlling and eliminating malaria, the disease still remains a major public health problem. It was estimated that 228 million cases of malaria occurred worldwide in 2018 (decreased from 231 million cases in 2017), and that there were 405,000 deaths in 2018 compared to 416,000 in 2017. Most malaria cases (93%) and deaths (94%) occurred in the World Health Organization (WHO) Africa Region, with *Plasmodium falciparum* accounting for 99.7% of the cases [1]. Children under 5 years of age and pregnant women are the most at risk of malaria infection [2, 3]. In sub-Saharan Africa where moderate to high transmission of malaria occurs, an estimated 11 million out of 38 million (29%) pregnancies were exposed to malaria in 2018 [1]. Although often asymptomatic, *P. falciparum* infection in pregnancy is associated with unfavourable pregnancy outcomes such as stillbirth, low birth weight (LBW), pre-term delivery, abortion and maternal anaemia [4–6]. Prevalence of malaria in pregnant women peaks in the second trimester. Malaria in pregnancy is also a useful marker for malaria surveillance at community level [7, 8] with common risk factors being a primagravida and being young [2, 9, 10].

As the burden of malaria varies in different geographical locations and population groups, surveillance of malaria cases has been recommended by WHO to identify areas or population groups most affected by malaria so that the necessary resources and interventions can be targeted at these groups [11], and their impact monitored and evaluated [12]. The general epidemiology of malaria has been comprehensively described for different age groups in the area of Ghana in which this study was undertaken [13] but information on the characteristics of malaria in pregnant women is sparse.

This study aimed at describing the prevalence and risk factors for malaria and anaemia among pregnant women living in an area of high malaria transmission in the middle belt of Ghana, prior to administration of intermittent preventive treatment with sulfadoxine-pyrimethamine (IPTp-SP).

## Methods

### Study area

**Fig. 1:**
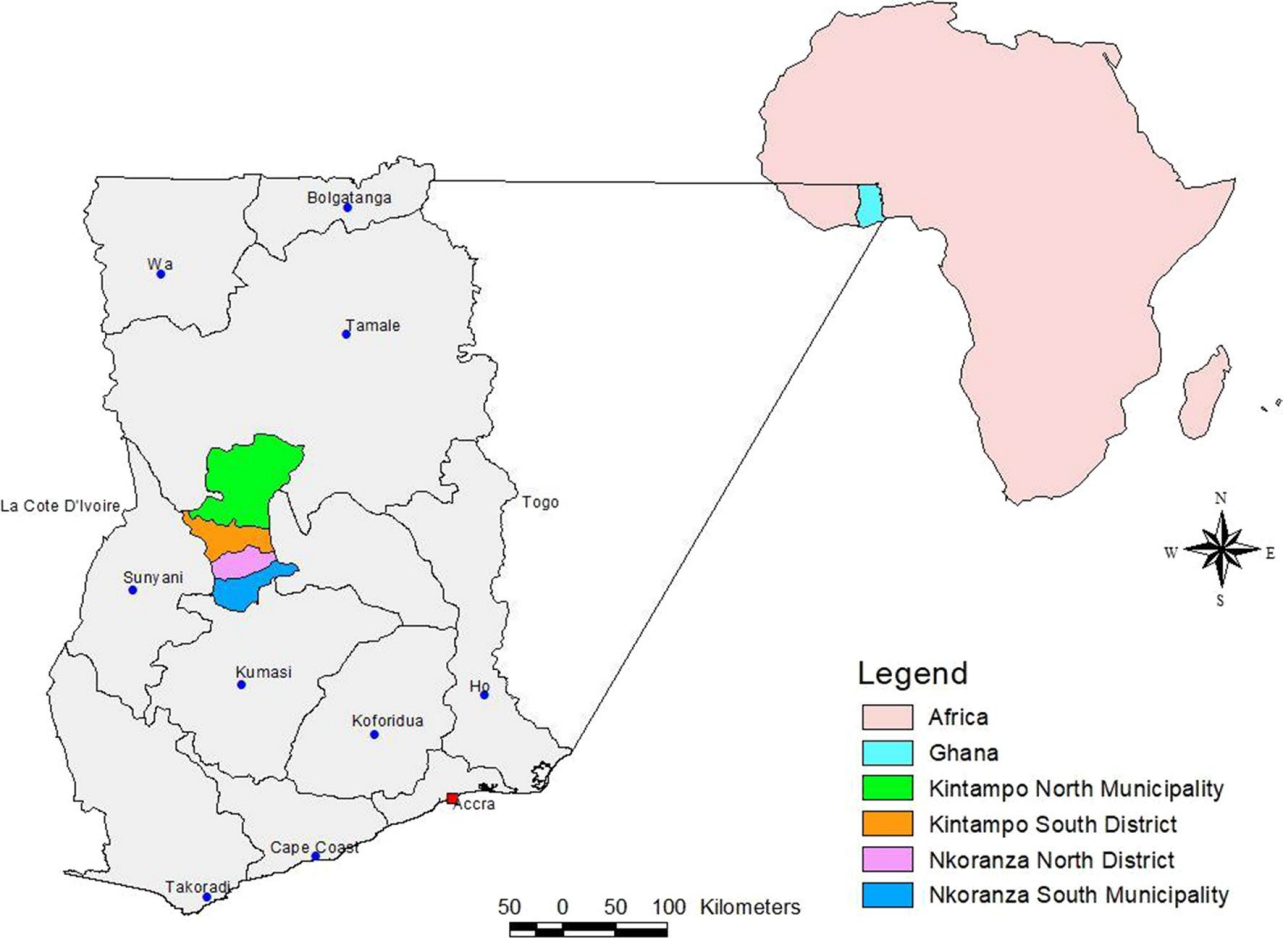
Map showing the study areas in the middle belt of Ghana

This study was conducted in four adjoining administrative areas, the Kintampo North Municipality, Kintampo South District, Nkoranza South Municipality, and Nkoranza North District, located in the Bono East Region, within the forest-savannah, transitional ecological zone in the middle belt of Ghana (Fig. 1). The Kintampo districts have a surface area of 7162 sq km, an approximate resident population of 150,000 and 4000 births per year [14]. Mean monthly temperature ranges from 18 to 38 °C while rainfall averages 1250 mm per annum. The main vectors for transmission of malaria are *Anopheles gambiae* and *Anopheles funestus* and the transmission is perennial, but peaks between April and October. The annual entomological inoculation rate was 269 infective bites per person per year in 2005 [13, 15], but is currently likely to be less. The Nkoranza districts have an approximate surface area of 2400 sq km and resident population of 130,000.

### Study design

This was an analysis of baseline data collected from pregnant women enrolled into a cohort study evaluating the effectiveness and safety of four or more doses of IPTp-SP. Eligibility criteria included pregnant women of any gravidity who attended the antenatal care (ANC) clinic for the first time, who had a gestational age 13–22 weeks, were aged 18 years or above, were known negative for human immunodeficiency virus (HIV) infection, and were willing to participate in the study (demonstrated by signing or thumb-printing an informed consent form).

### Enrolment of participants

Enrolment of study participants was done at ANC clinics within the study area from July 2017 to March 2019. A general introduction on the nature of the study was given to the pregnant women by the nursing staff at the ANC during their health talks. An initial screening was done to determine eligibility. For those meeting the inclusion criteria, a member of the study team then explained the study further including what an informed consent is, why the study was being done, what it involved in terms of benefits, risks, compensation, confidentiality, right to refuse to be part of the study, and the right to still receive all routine ANC services. Following collection of informed consent, eligible study participants had a questionnaire completed which included information on demographics, obstetric history, socio-economic status (SES), malaria symptoms, anti-malarial drug use, insecticide-treated bed net (ITN) use, and use of other malaria vector preventive measures.

### Sample collection

Prior to commencement of IPTp-SP, a blood sample (2 mL) was collected by a trained member of the study team using sterile venepuncture and transferred into a K_2_-EDTA tube (BD, Berkshire, UK) for a full blood count. Samples were kept in a transport box containing cold packs and transported to the laboratory in Kintampo for analysis.

### Haematological analysis

Haemoglobin estimations were performed using K_2_-EDTA anticoagulated blood on a validated and calibrated Horiba-ABX Micros 60 or Pentra 60 C+ haematology analyser (Horiba-ABX, Montpellier, France) within 8 h of blood draw in the Haematology Unit of the Kintampo Health Research Centre (KHRC) Clinical Laboratory. In addition to regular user maintenance, routine servicing of the analysers was performed quarterly. Three levels of quality control samples (low, normal and high) were tested daily prior to analysis of study samples to ensure the reliability of results. The laboratory also participated in monthly external quality assessment schemes organized by the UK National External Quality Assessment Scheme (UK NEQAS, Watford, UK).

### Malaria microscopy

Preparation and reading of malaria blood slides was done in the Parasitology Unit of KHRC Clinical Laboratory, as described by Swysen et al. [16] and WHO guidelines for preparation, staining and reading of malaria blood slides were followed [17]. Briefly, 6 µL and 2 µL of blood was used to prepare a thick smear and a thin smear, respectively, on the same slide using a template and the smears were then air-dried. Thin smears were fixed with absolute methanol, allowed to air-dry and both thick and thin smears were stained with 10% Giemsa stain (BDH Laboratory Supplies, Poole, Dorset, UK) in phosphate-buffered water (pH 7.2) for 10 min. Stained smears were air-dried and thick films were examined using an Olympus CX21 Microscope (Olympus, Tokyo, Japan) with × 10 eyepieces and ×100 oil immersion objectives to determine the presence, species and stages of any parasites that were present. The thin smear was used for confirming species and counting of parasites if more than 100 parasites were seen in the first field in the thick smear. Parasite density (parasites per µL of blood) was calculated using participants’ white blood cell (WBC) or red blood cell (RBC) count determined on an automated haematology analyser. A minimum of 100 fields were examined before a slide was recorded as negative. Each slide was independently examined by two microscopists who were certified by South Africa’s National Institute for Communicable Diseases (NICD) and/or the World Health Organization. The final result was determined as described by Swysen et al. [16]. As part of quality assurance, each batch of the 10% Giemsa stain was quality controlled by staining a known positive and negative smear prior to staining smears of study participants. Only certified malaria microscopists were allowed to read and report results for the study.

### Sample size calculation

A total of 1655 pregnant women who were enrolled in the main study designed to evaluate the impact of four or more doses of SP on placental malaria, LBW and anaemia were included in this cross-sectional study. With a malaria prevalence of 17.6% reported among pregnant women at outpatient departments (OPDs) in Ghana by the National Malaria Control Programme [18], the study estimated the prevalence of malaria with a precision of 1.8%.

### Outcome definitions

Clinical malaria was defined as parasitaemia and an axillary temperature ≥ 37.5 °C or a history of fever within the past 48 h, while asymptomatic malaria was defined as any level of parasitaemia without fever. The level of parasitaemia was classified as very low (1–199 parasites/µL), low (200–999 parasites/μL), medium (1000–10,000 parasites/μL), and high (> 10,000 parasites/μL) after modification of published cut-offs to create the very low category [19, 20]. Anaemia was defined as a haemoglobin concentration < 11.0 g/dL, and sub-divided as severe (< 7.0 g/dL), moderate (7.0–9.9 g/dL), and mild (10.0–10.9 g/dL) [21]. ITN use was defined as sleeping under a net the previous night, and use of other malaria vector preventive measures defined as usage within the past 7 days.

### Data management and statistical analysis

Enrolment, haematology and parasitology data were entered into pre-coded questionnaires and checked for completeness. Completed questionnaires were double-entered and verified using a database designed with CSharp application as front end and SQL Server as back end. Data processing and statistical analysis were carried out using Stata 14 (StataCorp, College Station, USA). Principal components analysis (PCA) was used to derive a wealth index variable (SES) based on each woman’s household assets and characteristics. Socio-demographic, obstetric and malaria vector prevention characteristics of all women enrolled in the study were summarized.

Wilcoxon rank-sum test was used to assess the association between parasite density and age (< 25 or ≥ 25) and gestational age (< 18 weeks or ≥ 18 weeks). The association of gravidity (primagravid, secundigravid, or multigravid) with parasite density was explored using Kruskal–Wallis test. The prevalence of malaria parasitaemia was computed together with its 95% confidence interval. Univariate and multivariate logistic regression was used to study the association between socio-demographic, obstetric and malaria vector prevention characteristics and the risk of malaria parasitaemia. Variables in the univariate analysis with a p-value < 0.15 were included in a multivariate logistic regression model. The interaction of age and gravidity on the risk of malaria parasitaemia was also explored.

Haemoglobin concentration in participants who had malaria parasitaemia was compared to those who did not have malaria parasitaemia using Wilcoxon rank-sum test. The prevalence of anaemia was further classified into mild, moderate and severe, and reported with 95% confidence intervals. Univariate and multivariate logistic regression was used to assess the association of socio-demographic, obstetric and malaria vector prevention characteristics of study participants with the risk of anaemia. Similar to the approach used in the analysis of malaria parasitaemia, variables from the univariate analysis with a p-value < 0.15 were included in a multivariate logistic regression model. The interaction of age and gravidity on the risk of anaemia was also explored. In all multivariate models, significance was established at a 5% level.

## Results

### Socio-demographic, obstetric, clinical, and malaria vector prevention characteristics of study participants at enrolment

A total of 1655 participants were eligible for inclusion in the study. Malaria microscopy results were available for 1647 (99.5%) participants, while haemoglobin results were available for 1465 participants (88.5%); samples from 182 were not adequate for haemoglobin estimation. Mean age (± SD) of the participants was 27.4 (± 6.2) years, with nearly half (49.6%) of them aged 25–34 years and the mean gestational age at enrolment (± SD) was 16.7 (± 4.3) weeks, with about two-thirds (67.4%) of women having a gestational age ≤ 18 weeks. Primigravidae and secundigravidae formed 21.0% (n = 355) and 23.7% (n = 392) of the participants, respectively. About one-fifth, 20.9% (n = 346) had completed high school or higher education.

Almost 90.0% (1488/1655) of participants reported possessing an ITN, of which 12.1% (180/1488) were reported torn. Reported ITN use among all women and those possessing an ITN was 66.2% (1095/1655) and 73.6% (1095/1488), respectively. About one-fifth of the participants reported using a mosquito coil within the last 7 days (Table 1). The main clinical complaints at enrolment were abdominal pain (7.9%, n = 131), bodily pains (7.9%, n = 130), loss of appetite (6.6%, n = 109), fever (6.2%, n = 106), drowsiness (4.8%, n = 80), vomiting (4.6%, n = 76), and nausea (4.2%, n = 69).

**Table 1:** Socio-demographic characteristics of pregnant women at enrolment

Characteristics	Number of participants (N = 1655)	Percentage of participants (%)
Maternal age (years)		
≤ 24	587	35.5
25–34	821	49.6
≥ 35	247	14.9
Highest educational level		
None	482	29.1
Primary school	285	17.2
Junior high school	542	32.8
Senior high school	247	14.9
Tertiary	99	6.0
Household SES		
Most poor	331	20.0
More poor	330	19.9
Poor	332	20.1
Less poor	331	20.0
Least poor	331	20.0
Marital status		
Married	1223	73.9
Single, unmarried	432	26.1
Religion		
Christian	1216	73.5
Muslim	365	22.0
Others	74	4.5
Profession		
Professional teacher, nurse, accountant, administrator	77	4.7
Clerical/Secretarial	6	0.4
Trader/food seller/business woman	513	31.0
Seamstress, hairdresser, etc.	259	15.7
Farmer/labourer/domestic worker	350	21.1
Other	56	3.4
No profession	394	23.7
Maternal BMI (kg/m^2^)		
< 18.5	51	3.1
18.5–24.9	986	59.6
25.0–29.9	408	24.7
≥ 30.0	133	8.0
Missing data	77	4.6
Malaria vector prevention		
ITN possession	1488	89.9
ITN use	1095	66.2
ITN torn	180	10.9
Mosquito coil use	361	21.8
Insecticide spray use	194	11.7
Commercial repellent use	36	2.2
Traditional repellent use	4	0.2

### Malaria prevalence, species and density

Overall prevalence of malaria parasitaemia was 20.4% (95% CI 18.5–22.4%) and the prevalence of clinical malaria was 1.5% (95% CI 1.0–2.2%). The prevalence of parasitaemia was higher in women aged < 25 years (31.2%; 95% CI 27.6–35.0%) compared to those ≥ 25 years (14.5%, 95% CI 12.5–16.8%), and also higher in primigravidae (31.0%, CI 26.4–36.0%) compared to multigravidae (15.1%, 95% CI 12.9–17.6%). *Plasmodium falciparum* was the only species identified among the *Plasmodium* isolates. Overall geometric mean parasite density (GMPD) was 442 parasites per µL (95% CI 380–515). Density was also higher in women aged < 25 years (628 parasites per µL, 95% CI 511–772) compared to those ≥ 25 years (293 parasites per µL, 95% CI 238–362), and in primigravidae (721 parasites per µL, 95% CI 554–938) compared with multigravidae (295 parasites per µL, 95% CI 234–372) (Table 2). Among pregnant women with malaria parasites, more than three-quarters had parasite density below 1000 parasites/µL of blood. The proportion of women with very low, low, medium, and high density parasitaemia were 31.1% (95% CI 26.3–36.2%), 47.0% (95% CI 41.7–52.4%), 18.9% (95% CI 15.1–23.5%), and 3.0% (95% CI 1.6–5.4%), respectively.

**Table 2:** Malaria prevalence and geometric mean parasite density by age, gestational age, gravidity, and enrolment location

	N	Overall parasitaemia	Parasite density	
			GMPD (95% CI)	p-value
Total	1647	20.4 (18.5–22.4)	442 (380–515)	
Age				
< 25	582	31.2 (27.6–35.0)	628 (511–772)	< 0.001
≥ 25	1065	14.5 (12.5–16.8)	293 (238–362)	
Gestation age, weeks^a^				
< 18	1111	19.9 (17.7–22.3)	427 (352–517)	0.438
≥ 18	518	21.7 (18.3–25.4)	480 (370–622)	
Gravidity				
Primigravidae	353	31.0 (26.4–36.0)	721 (554–938)	< 0.001
Secundigravidae	391	23.2 (19.3–27.7)	451 (338–600)	
Multigravidae	903	15.1 (12.9–17.6)	295 (234–372)	

^a^Gestation age missing for 18 participants

### Factors associated with malaria parasitaemia

In the unadjusted analyses, age, education, household SES, marital status, profession, gravidity, and ITN use were associated with risk of malaria parasitaemia (Table 3). In the multivariate analysis, women who reported using an ITN were 1.43 times likely to have parasitaemia compared to those who did not. Women ≥ 25 years of age were 0.57 times less likely than those < 25 years of age, multigravidae were 0.50 times less likely compared to primagravidae, traders or food sellers were 0.67 times less likely than women with no profession, women educated to high school or above were 0.53 times less likely compared to those with no education and the least poor were 0.34 times less likely compared to the most poor to have malaria parasitaemia (Table 3).

**Table 3:** Factors associated with malaria parasitaemia at first antenatal clinic attendance in the middle belt of Ghana

Factors	Malaria Parasitaemia			COR	95% CI	Overall p-value	AOR	95% CI	Overall p-value
	Total (N = 1647) n	Present (N = 338) n (%)	Absent (N = 1309) n (%)						
Age, years									
< 25	582	183 (31.4)	399 (68.6)	1		< 0.001	1		0.001
≥ 25	1065	155 (14.6)	910 (85.4)	0.37	0.29-0.47		0.57	0.41–0.79	
Gestation age, weeks^a^									
< 18	1111	222 (20.0)	889 (80.0)	1		0.394			
≥ 18	518	113 (21.8)	405 (78.2)	1.12	0.87–1.44				
Education									
No education	479	108 (22.6)	371 (77.4)	1		0.009	1		0.041
Primary	285	66 (23.2)	219 (76.8)	1.04	0.73–1.47		0.87	0.60–1.26	
Junior High	540	115 (21.3)	425 (78.7)	0.93	0.69–1.25		0.73	0.52–1.03	
High School or above	343	49 (14.3)	294 (85.7)	0.57	0.40–0.83		0.53	0.33–0.83	
HH SES									
Most poor	330	105 (31.8)	225 (68.2)	1		< 0.001	1		< 0.001
More poor	329	65 (19.8)	264 (80.2)	0.53	0.37–0.75		0.42	0.33–0.71	
Poor	330	74 (22.4)	256 (77.6)	0.62	0.44–0.88		0.61	0.42–0.90	
Less poor	327	59 (18.0)	268 (82.0)	0.47	0.33–0.68		0.51	0.34–0.78	
Least poor	331	35 (10.6)	296 (89.4)	0.25	0.17–0.39		0.34	0.21–0.54	
Marital status									
Married	1218	215 (17.7)	1003 (82.4)	1		< 0.001	1		0.102
Not married	429	123 (28.7)	306 (71.3)	1.88	1.45–2.42		1.30	0.95–1.78	
Profession									
No profession	392	107 (27.3)	285 (72.7)	1		< 0.001	1		0.213
Professional teacher, clerical, secretary	83	5 (6.0)	78 (94.0)	0.17	0.07–0.43		0.44	0.16–1.20	
Trader, food seller	510	70 (13.7)	440 (86.3)	0.42	0.30–0.59		0.67	0.46–0.97	
Seamstress, hairdresser,	258	57 (22.1)	201 (77.9)	0.76	0.52–1.09		0.88	0.59–1.32	
Farmer/labourer	348	83 (23.9)	265 (76.1)	0.83	0.60–1.16		0.88	0.60–1.29	
Other	56	16 (28.6)	40 (71.4)	1.07	0.57–1.98		1.08	0.60–2.09	
Religion									
Christian	1210	255 (21.1)	955 (78.9)	1		0.392			
Muslim	364	66 (18.1)	298 (81.9)	0.83	0.61–1.12				
None/others	73	17 (23.3)	56 (76.7)	1.14	0.65–1.99				
Gravidity									
Primigravidae	353	110 (31.2)	243 (68.8)	1		< 0.001	1		0.002
Secundigravidae	391	91 (23.3)	300 (76.7)	0.67	0.48–0.93		0.78	0.55–1.11	
Multigravidae	903	137 (15.2)	766 (84.8)	0.40	0.30–0.53		0.50	0.33–0.74	
ITN use									
No	559	102 (18.3)	457 (81.8)	1		0.100	1		
Yes	1088	236 (21.7)	852 (78.3)	1.24	0.96–1.61		1.43	1.09–1.89	0.010

HH SES: Household socio-economic status; ITN: Insecticide-treated bednet

^a^Missing for 18 participants

### Effect of the interaction between age and gravidity on the prevalence of malaria parasitaemia

**Fig. 2:**
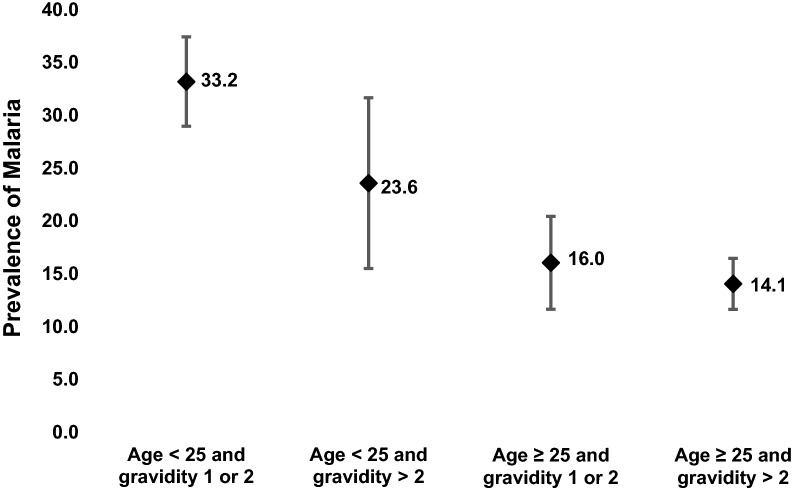
Effect of interaction between age and gravidity on prevalence of malaria parasitaemia

Figure 2 shows the effect of the interaction between age and gravidity on the prevalence of malaria parasitaemia. Prevalence was highest (33.2%) in primi- or secundigravidae aged < 25 years old and lowest (14.1%) in multigravidae aged ≥ 25 years. Compared to multigravidae aged ≥ 25 years, malaria parasataemia prevalence was 3.04 times higher (95% CI 2.31–4.01, p < 0.001) in primigravidae aged < 25 years and 1.89 times higher (95% CI 1.16–3.08, p = 0.011) in those ≥ 25 years, respectively. There was, however, no significant difference between multigravid women aged < 25 years and those ≥ 25 years (OR 1.17; 95% CI 0.80–1.71, p = 0.424).

### Prevalence of anaemia among pregnant women

**Fig. 3:**
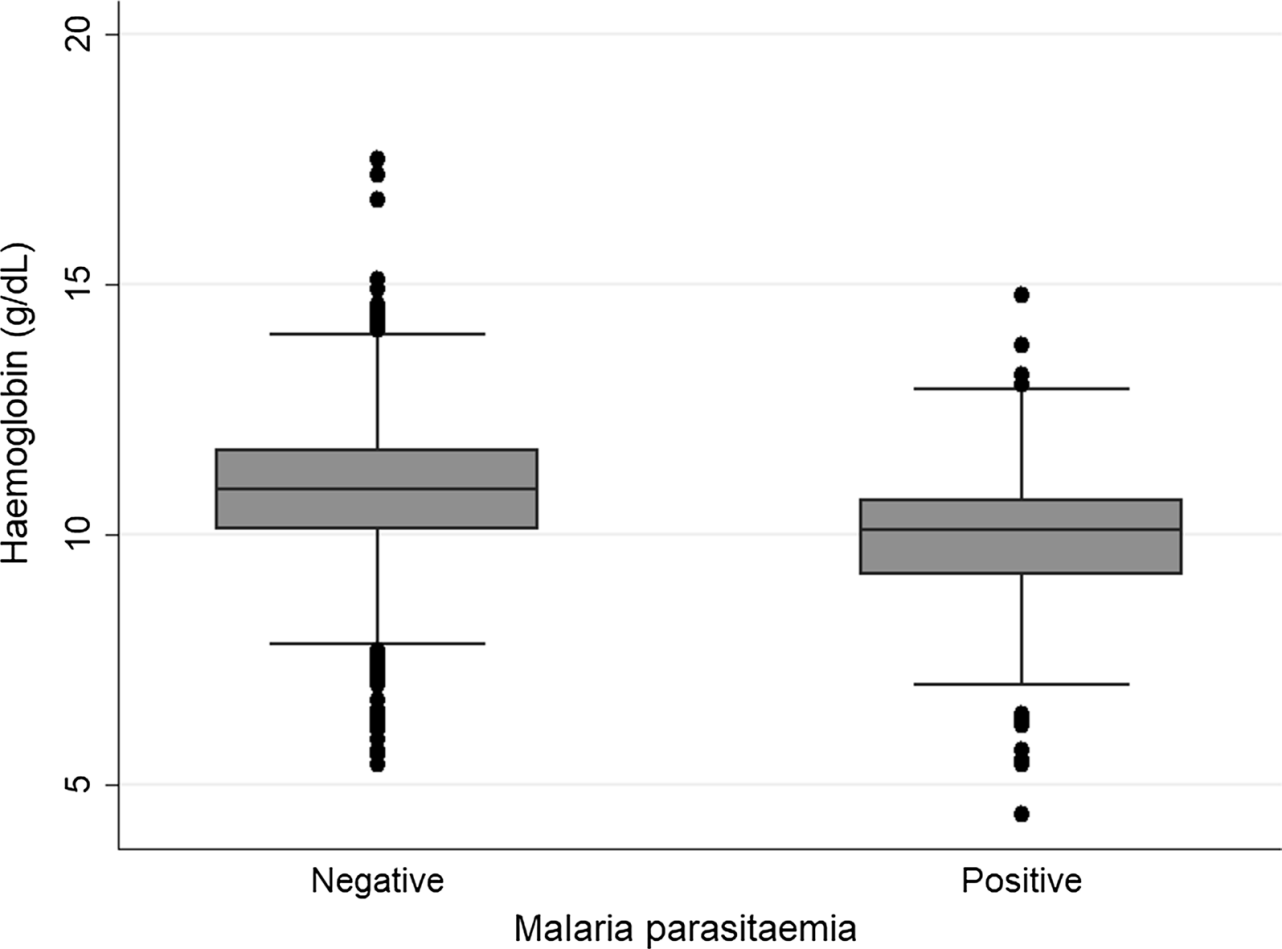
Haemoglobin concentration by malaria parasitaemia status

Mean (± SE) haemoglobin concentration for the pregnant women at first ANC visit was 10.7 g/dL (± 0.04). Among women with malaria parasitaemia, mean haemoglobin concentration was significantly lower than in those without malaria parasitaemia (9.9 g/dL vs 10.9 g/dL; p-value < 0.001) (Fig. 3). Overall prevalence of anaemia (haemoglobin < 11.0 g/dL) and severe anaemia (haemoglobin < 7.0 g/dL) were 56.0% (95% CI 53.4–58.5%) and 1.4% (95% CI 0.9–2.2%), respectively (Table 4).

**Table 4:** Prevalence of different degrees of anaemia among pregnant women in the middle belt of Ghana

Anaemia status (haemoglobin level)	Frequency (N = 1465)	Prevalence (%)	95% CI
Non-anaemic (≥ 11.0 g/dL)	645	44.0	41.5–46.6
Anaemic, all forms (< 11.0 g/dL)	820	56.0	53.4–58.5
Mild (10.0–10.9 g/dL)	428	29.2	26.9–31.6
Moderate (7.0–9.9 g/dL)	371	25.3	23.2–27.6
Severe (< 7.0 g/dL)	21	1.4	0.9–2.2

### Factors associated with anaemia among pregnant women

In the univariate analysis, age, gestational age, education, household SES, marital status, profession, gravidity, and malaria parasitaemia status were associated with risk of anaemia (Table 5). In the multivariate logistic regression analysis, the risk of anaemia was 0.61 times lower in multigravidae compared to primagravidae. Women with gestational age ≥18 weeks were 2.24 times more likely to have anaemia compared to those aged < 18 weeks, and unmarried women were 1.44 times likely to be anaemic compared to the married women. Women who had parasitaemia up to 999 parasites/µL were 3.49 times likely to have anaemia, whiles those with parasitaemia ≥ 1000 parasites/µL were 4.05 times more likely to have anaemia, compared to those without parasitaemia (Table 5).

**Table 5:** Factors associated with anaemia at first antenatal clinic attendance in the study area

Factors	Anaemia			COR	95% CI	p-value	AOR	95% CI	p-value
	Total (N = 1465) n	Present (N = 820) n (%)	Absent (N = 645) n (%)						
Age, years									
< 25	523	337 (64.4)	186 (35.6)	1		< 0.001	1		0.981
≥ 25	942	483 (45.2)	459 (48.7)	0.58	0.47–0.72		1.00	0.74–1.34	
Gestation age, weeks									
< 18	986	491 (49.8)	495 (50.2)	1		< 0.001	1		< 0.001
≥ 18	464	317 (68.3)	147 (31.7)	2.17	1.72–2.74		2.24	1.75–2.86	
Education									
No education	426	252 (59.2)	174 (40.9)	1		0.121	1		0.415
Primary	249	139 (55.8)	110 (44.2)	0.87	0.64–1.20		0.80	0.57–1.13	
Junior high	486	276 (56.8)	210 (43.2)	0.91	0.70–1.18		0.85	0.62–1.15	
High school or above	304	153 (50.3)	151 (49.7)	0.70	0.52–0.94		0.74	0.50–1.09	
HH SES									
Most poor	290	180 (62.1)	110 (37.9)	1		< 0.001	1		0.024
More poor	297	188 (63.3)	109 (36.7)	1.05	0.75–1.47		1.27	0.88–1.83	
Poor	291	169 (58.1)	122 (41.9)	0.85	0.61–1.18		1.00	0.69–1.46	
Less poor	291	151 (51.9)	140 (48.1)	0.66	0.47–0.92		0.84	0.57–1.24	
Least poor	296	132 (44.6)	164 (55.4)	0.49	0.35–0.68		0.69	0.46–1.03	
Marital status									
Married	1075	563 (52.4)	512 (47.6)	1		< 0.001	1		0.015
Not married	390	257 (65.9)	133 (34.1)	1.76	1.38–2.24		1.44	1.07–1.92	
Profession									
No profession	354	220 (62.2)	134 (37.9)	1		< 0.001	1		0.568
Professional teacher, clerical, secretary, …	78	31 (39.7)	47 (60.3)	0.40	0.24–0.66		0.85	0.47–1.52	
Trader, food seller	442	227 (51.4)	215 (48.6)	0.64	0.48–0.85		1.04	0.75–1.44	
Seamstress, hairdresser,	233	123 (52.8)	110 (47.2)	0.68	0.49–0.95		0.80	0.55–1.16	
Farmer/labourer	311	188 (60.5)	123 (39.6)	0.93	0.68–1.27		1.07	0.74–1.55	
Other	47	31 (66.0)	16 (34.0)	1.18	0.62–2.24		1.28	0.64–2.55	
Religion									
Christian	1077	589 (54.7)	488 (45.3)	1		0.184			
Muslim	324	190 (58.6)	134 (41.4)	1.17	0.91–1.51		–	–	–
Others	64	41 (64.1)	23 (35.9)	1.48	0.87–2.50		–	–	–
Gravidity									
Primigravidae	327	218 (66.7)	109 (33.3)	1		< 0.001	1		0.023
Secundigravidae	337	195 (57.9)	142 (42.1)	0.69	0.50–0.94		0.79	0.56–1.11	
Multigravidae	801	407 (50.8)	394 (49.2)	0.52	0.39–0.68		0.61	0.42–0.87	
ITN use									
No	964	532 (55.2)	432 (44.8)	1		0.401	–	–	–
Yes	501	288 (57.5)	213 (42.5)	0.91	0.73–1.13		–	–	–
Malaria Parasitaemia									
None	1182	592 (50.1)	590 (49.9)	1		< 0.001	1		< 0.001
Low/very low	209	166 (79.4)	43 (20.6)	3.85	2.70–5.48		3.49	2.41–5.05	
Medium/high	74	62 (83.8)	12 (16.2)	5.15	2.75–9.65		4.05	2.11–7.78	

HH SES: Household socio-economic status; ITN: Insecticide-treated bednet

### Interaction between age and gravidity on prevalence of anaemia among pregnant women

**Fig. 4:**
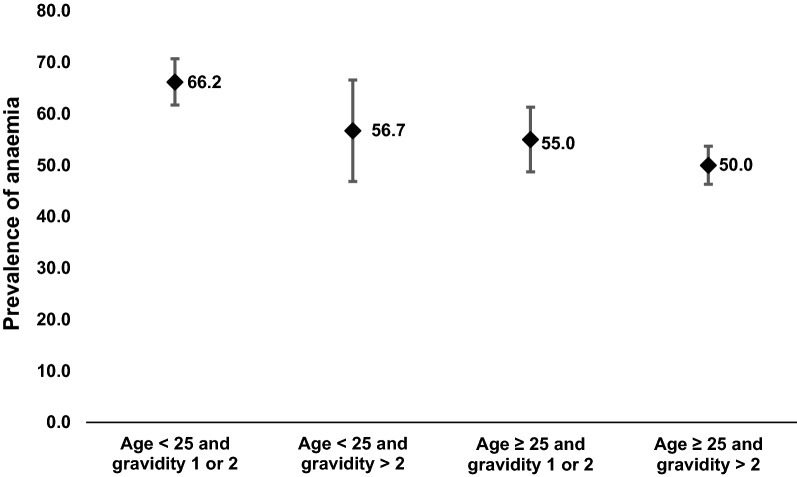
Effect of interaction between age and gravidity on prevalence of anaemia

The effect of the interaction between age and gravidity on prevalence of anaemia is shown in Fig. 4. Prevalence of any anaemia was highest (66.2%) in primi- or secundigravidae aged < 25 years and least (50.0%) in multigravid pregnant women aged ≥ 25 years. Compared to multigravid pregnant women aged ≥ 25 years, anaemia prevalence was significantly higher (p < 0.001) in primi- or secundigravidae aged < 25 years (COR 1.96; 95% CI 1.53–2.51). There was no significant difference in the prevalence of anaemia between multigravidae < 25 years and ≥ 25 years.

## Discussion

This study observed a high prevalence of malaria parasitaemia among pregnant women during their first ANC visit in the middle belt of Ghana where malaria transmission is high and perennial. The overall malaria parasitaemia prevalence in this study population (20.4%) is similar to that reported from Hohoe municipality in Ghana (20.3%) but is much higher than that reported from other sites in Ghana: Madina, Accra (5.0%) [22] and Ejisu Buabeng and Sekyere East Districts (10.7%) [23]. The prevalence of parasitaemia seen in the study area is comparable to that reported from elsewhere in Africa: 22.4% in Mount Cameron, Cameroon [24], 19.6% in Blantyre, Malawi [25] and in Mali [10], however, there were reports of higher prevalence of parasitaemia in Nchelenge, Zambia (31.8%) [2] and Navrongo, Ghana (42.0%) [10], respectively. Prevalence of clinical malaria reported in this study (1.5%) was lower compared to findings reported in Malawi [25] and Cameroon [24]. The marked variation in malaria prevalence across the various studies is likely to be due to differences in malaria transmission intensity, malaria prevention measures used, rainfall, and environmental conditions [26, 27]. Finding higher malaria parasitaemia prevalence in younger primigravidae, which decreased with increasing age and gravidity, is consistent with several other studies [10, 27, 28].

The overall GMPD reported in this study (442 parasites/µL) is similar to that among pregnant women in Mount Cameroon, Cameroon [24], but much lower than that reported by other authors [2, 28]. This could be due to differences in transmission intensities and different levels of immunity in different locations. It could also partly be due to the use of participants’ own absolute WBC counts in this study rather than the assumed WBC count of 8000 per µL of blood, which was used in many of these studies in calculating parasite density. The use of assumed 8000 WBCs per µL of blood has been reported to result in overestimation of malaria parasite density several-fold among pregnant women [29] and children [30]. Although only *P. falciparum* was detected using microscopy in this study, this is not surprising. The use of the PCR technique may have resulted in detection of other species, as a study of non-falciparum malaria in northern Ghana showed a prevalence of 0.96% [31]. The findings in this study also indicated that the risk of malaria parasitaemia was higher in women in their first pregnancy, younger women, or those of lower SES or education. These observations are consistent with other published studies in Gabon [32], Ghana [10, 28] and Zambia [2],

Surprisingly, this study found reported ITN use to be associated with higher risk of malaria parasitaemia in multivariate analysis, which is contrary to what has been reported in many studies [33, 34] where regular use of ITN by pregnant women was associated with protection against malaria. The finding on ITN use in this study is, however, consistent with a report from Navrongo, Ghana [10]. This observation, however, does not suggest ITN use does not protect against malaria. The state of the ITN used would also need to be taken into consideration, as about 12% of participants in this study reported the nets in which they slept were torn. Current ITN use could also be a marker of a person living in an area with high mosquito biting rates making them more likely to use an ITN than a person living in an area where mosquito bites and malaria were infrequent. [10].

The findings on the overall prevalence of anaemia among pregnant women in this study is higher than that reported from the Sunyani Municipality of Ghana [35], similar to that from Cameroon (53.4%) [36], but much lower than that reported from other districts in Ghana: 64.8% [37] and 70.0% [28], and Nigeria (71.3%) [38]. The factors strongly associated with anaemia in this study (i.e., being primagravid, gestational age at first ANC visit, having malaria parasitaemia) are similar to those reported in many other studies in different geographical locations [28, 35, 38].

The strength of this study is that it is adequately powered to provide accurate information on the high prevalence of malaria in pregnant women in the study area and the need for effective measures of control and for monitoring their progress. The study, however, has some limitations. Firstly, malaria microscopy has been shown to underestimate malaria parasite prevalence compared to molecular methods due to the presence of sub-microscopic levels of parasitaemia. However, quality-assured malaria microscopy remains the gold standard for diagnosis and surveillance. Strict adherence to standard operating procedures was ensured and internationally certified microscopists used for the reading of malaria slides. Secondly, although helminth infections haemoglobinopathies have been identified as important risk factors of anaemia, stool examination and haemoglobin genotyping could not be performed during this study.

## Conclusions

The prevalence of *P. falciparum* infection during a first ANC visit in the middle belt of Ghana is high, with one out of five women infected and one out of three first-time pregnant women aged < 25 years infected. More than three-quarters of these infections were below 999 parasites/µL of blood. There is the need to strengthen existing malaria prevention strategies and also to employ targeted interventions to control malaria in the study area. This study provides indicators for regular monitoring of progress made towards malaria control or elimination.

## Data Availability

The datasets used and/or analysed during the current study are available from the corresponding author on reasonable request.

## Abbreviations

ANC: Antenatal care
AOR: Adjusted odds ratio
CI: Confidence interval
COR: Crude odds ratio
GMPD: Geometric mean parasite density
HH: Household
IPTp: Intermittent preventive treatment during pregnancy
ITN: Insecticide-treated bednet
K_2_-EDTA: Di-potassium ethylenediaminetetraacetic acid
KHRC: Kintampo Health Research Centre
LBW: Low birth weight
NICD: National Institute for Communicable Diseases
OPD: Out-Patient Department
PCA: Principal component analysis
RBC: Red blood cell
SD: Standard deviation
SES: Socio-economic status
SP: Sulphadoxine-pyrimethamine
UK NEQAS: United Kingdom National External Quality assessment Scheme
WBC: White blood cell
WHO: World Health Organization
